# New *Obolenskvirus* Phages Brutus and Scipio: Biology, Evolution, and Phage-Host Interaction

**DOI:** 10.3390/ijms25042074

**Published:** 2024-02-08

**Authors:** Peter V. Evseev, Mikhail M. Shneider, Lyubov V. Kolupaeva, Anastasia A. Kasimova, Olga Y. Timoshina, Andrey V. Perepelov, Anna M. Shpirt, Andrey A. Shelenkov, Yulia V. Mikhailova, Natalia E. Suzina, Yuriy A. Knirel, Konstantin A. Miroshnikov, Anastasia V. Popova

**Affiliations:** 1 Shemyakin-Ovchinnikov Institute of Bioorganic Chemistry, Russian Academy of Sciences, 117997 Moscow, Russia; mikhailshneider@gmail.com (M.M.S.); lalatimosha@gmail.com (O.Y.T.); kmi@bk.ru (K.A.M.); 2 State Research Center for Applied Microbiology and Biotechnology, City District Serpukhov, Moscow Region, 142279 Obolensk, Russia; melstryder@yandex.ru (L.V.K.); nastia-kasimova979797@mail.ru (A.A.K.); 3 Pirogov Russian National Research Medical University, 117997 Moscow, Russia; 4 N. D. Zelinsky Institute of Organic Chemistry, Russian Academy of Sciences, 119991 Moscow, Russia; perepel@ioc.ac.ru (A.V.P.); asyashpirt@gmail.com (A.M.S.); yknirel@gmail.com (Y.A.K.); 5 Central Scientific Research Institute of Epidemiology, 111123 Moscow, Russiamihailova@cmd.su (Y.V.M.); 6 Skryabin Institute of Biochemistry and Physiology of Microorganisms, Federal Research Center “Pushchino Center for Biological Research of the Russian Academy of Sciences”, Moscow Region, 142290 Pushchino, Russia; suzina_nataliya@rambler.ru

**Keywords:** *Acinetobacter baumannii*, bacteriophage, *Obolenskvirus*, phage evolution, tailspike depolymerase, capsular polysaccharide

## Abstract

Two novel virulent phages of the genus *Obolenskvirus* infecting *Acinetobacter baumannii*, a significant nosocomial pathogen, have been isolated and studied. Phages Brutus and Scipio were able to infect *A. baumannii* strains belonging to the K116 and K82 capsular types, respectively. The biological properties and genomic organization of the phages were characterized. Comparative genomic, phylogenetic, and pangenomic analyses were performed to investigate the relationship of Brutus and Scipio to other bacterial viruses and to trace the possible origin and evolutionary history of these phages and other representatives of the genus *Obolenskvirus*. The investigation of enzymatic activity of the tailspike depolymerase encoded in the genome of phage Scipio, the first reported virus infecting *A. baumannii* of the K82 capsular type, was performed. The study of new representatives of the genus *Obolenskvirus* and mechanisms of action of depolymerases encoded in their genomes expands knowledge about the diversity of viruses within this taxonomic group and strategies of *Obolenskvirus*–host bacteria interaction.

## 1. Introduction

The ability of bacterial viruses (bacteriophages or phages) to kill bacteria attracted the attention of researchers very soon after the discovery of phages by Frederick W. Twort in 1915 and Félix d’Hérelle in 1917 [1,2,3]. Phage therapy has important advantages, including low intrinsic toxicity, minimal disruption of normal microbiota, and the lack of cross-resistance with antibiotics [4]. The spread of antibiotic resistance and advances in molecular biology and virology have brought renewed attention to bacteriophage treatment in recent decades. Phage therapy could provide an effective treatment of nosocomial infections and would be a solution to the problems caused by the global spread of antimicrobial resistance [5].

Over the past few decades, *Acinetobacter baumannii* has emerged as one of the most troublesome nosocomial pathogens [6]. *A. baumannii* causes a wide range of hospital-acquired infections including respiratory tract, bloodstream, skin and soft tissue, urinary tract, and central nervous system infections and can be associated with high morbidity and mortality rates [6,7]. As a member of the «ESKAPE» (*Enterococcus faecium*, *Staphylococcus aureus*, *Klebsiella pneumoniae*, *Acinetobacter baumannii*, *Pseudomonas aeruginosa,* and *Enterobacter* species) group of pathogens, *A. baumannii* effectively escapes the effects of antibacterial drugs [8,9]. In this regard, the use of virulent bacteriophages, which are highly specific to their targets, is a promising approach to controlling the spread of multidrug-resistant *A. baumannii* strains.

In this work, we present a characterization of two novel phages, Brutus and Scipio, isolated on *A. baumannii* strains belonging to K116 and K82 capsular types, respectively. The phages are closely related to the viruses assigned to the genus *Obolenskvirus*. This genus is one of the largest groups of *A. baumannii* phages, which currently comprises more than thirty phages, with genomes deposited in NCBI GenBank [10]. The genomes of each *Obolenskvirus* phage encode one tailspike depolymerase that determines specificity to a certain *A. baumannii* K type. To date, the enzymatic activity of only one representative of the genus *Obolenskvirus* has been established—that of the K91(40)-specific phage AP22 [11]. Herein, the first characterization of the mechanism of action of a K82-specific enzyme encoded in the Scipio genome was made.

The thorough examination of Brutus and Scipio genomes, and the genomes of other *Obolenskvirus* phages, revealed that these viruses share similarities in genome organization and composition but also have differences in terms of their evolutionary history. Some of those differences most likely illustrate the theory of the modular evolution of phages and underline the role of horizontal transfer in phage evolution. Furthermore, some genomic features can be explained by their relatedness to phages that are capable of a temperate lifestyle. These observations are supported by proteomic phylogeny and pangenomic analysis. The analysis of core protein phylogeny and calculations of intergenomic similarity enable the suggestion of taxonomic updates that can better correspond to the genomic data.

## 2. Results

### 2.1. Phage Morphological Characteristics and Infection Parameters

**Figure 1:**
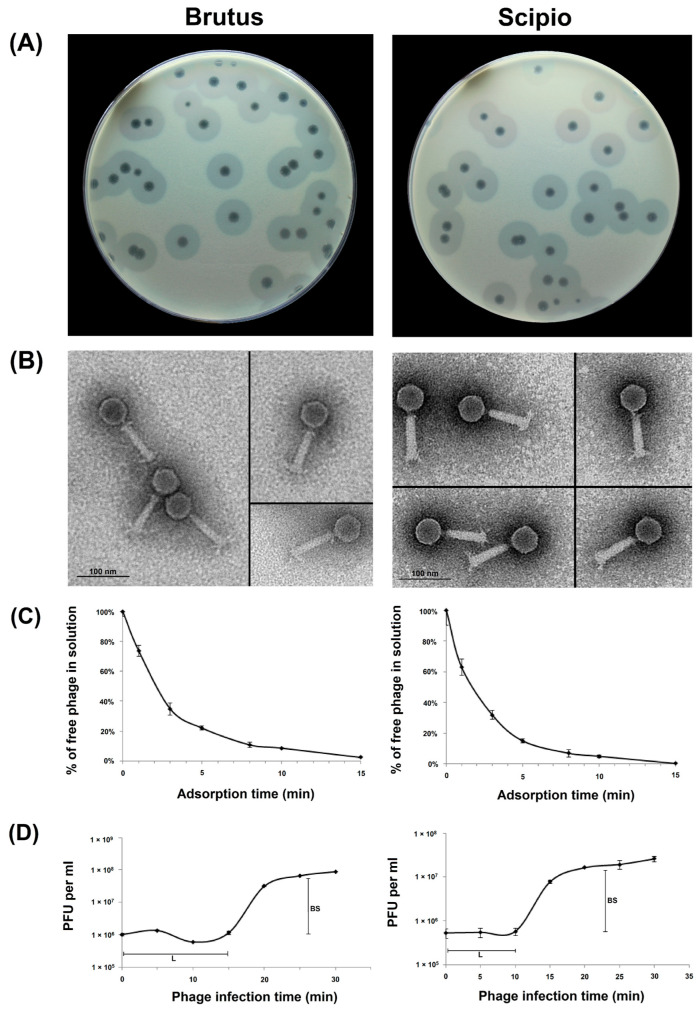
(**A**) Brutus and Scipio plaques with opaque haloes on *A. baumannii* MAR15-3273 (K116 capsular type) and *A. baumannii* LUH5534 (K82 capsular type), respectively, after 18–24 h incubation of plates at 37 °C. (**B**) Transmission electron micrographs of the phage particles. Staining with 1% uranyl acetate. The scale bar is 100 nm. (**C**) Adsorption assay of phages Brutus and Scipio on *A. baumannii* MAR15-3273 and *A. baumannii* LUH5534, respectively. (**D**) One-step growth curves of phages Brutus and Scipio on *A. baumannii* MAR15-3273 and *A. baumannii* LUH5534, respectively, with an indication of estimated burst sizes (BS) and latent periods (L). The results are the means and standard deviations from three independent experiments. PFUs: plaque-forming units.

Bacteriophages Brutus and Scipio were isolated from sewage samples collected, in 2018, from the Moscow region, in Russia, from lawns of *A. baumannii* MAR15-3273 and *A. baumannii* LUH5534, respectively. The structure of K82 capsular polysaccharide (CPS) produced by *A. baumannii* LUH5534 was established earlier [12]. The capsule biosynthesis gene cluster identified in the A*. baumannii* MAR15-3273 genome was found to be identical to the previously described locus of *A. baumannii* MAR-303 assigned to KL116 (GenBank accession number MK399425.1) [13]. On the host bacterial lawns, both phages form clear plaques with haloes (Figure 1A), indicating the presence of the depolymerizing activity of certain phage structures toward corresponding CPSs.

Phage particles’ morphology was examined by transmission electron microscopy. Brutus and Scipio were found to have icosahedral heads of 58.7 ± 3.4 nm and 59.3 ± 1.9 nm in diameter and contractile tails of about 93.2 ± 1.4 nm and 92.9 ± 1.8 nm in length with distinguishable tailspikes, respectively (Figure 1B). Interestingly, both phages also have a short, thin neck between the head and the tail and what are presumed to be collar structures, which are visible in some electron micrographs.

The infection process for both phages was investigated by estimating adsorption efficiency (Figure 1C) and one-step growth experiments (Figure 1D). It was shown that more than 85% of Scipio and almost 80% of Brutus phage particles adsorbed to the host cells within 5 min and exhibited adsorption constants of 1.9 × 10^−9^ mL/min and 1.5 × 10^−9^ mL/min for *A. baumannii* LUH5534 and *A. baumannii* MAR15-3273 host cells for a period of 5 min, respectively. The latent periods for Scipio and Brutus were 10 and 15 min, and the burst sizes were approximately 45.5 ± 3.7 and 75.0 ± 5.3 particles per infected cell, respectively (Figure 1D).

### 2.2. General Genome Features

**Figure 2:**
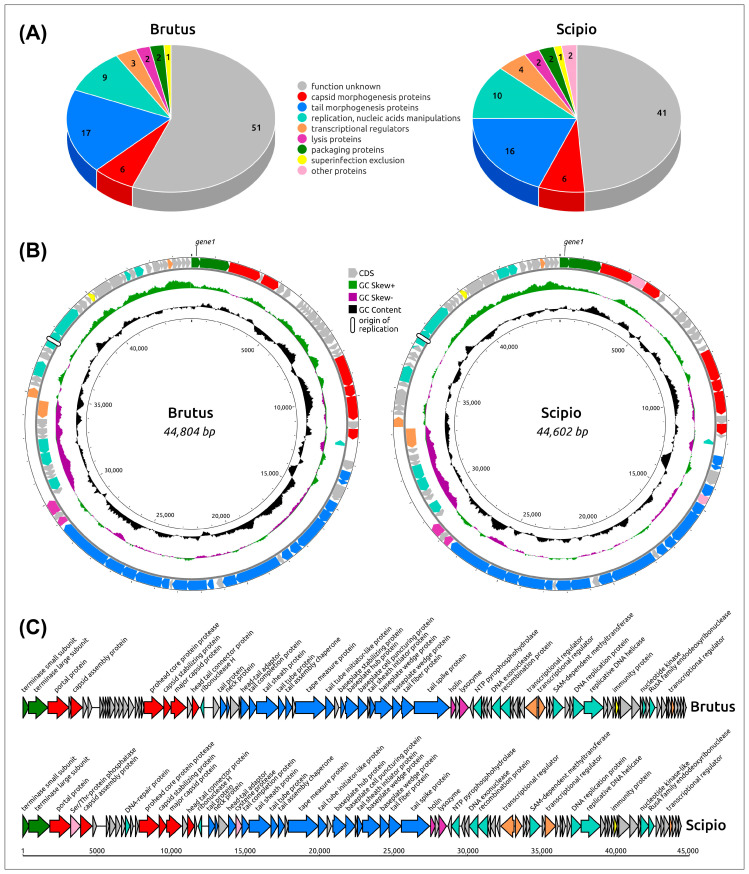
(**A**) Functional assignment of predicted genes in the genomes of phages Brutus and Scipio. (**B**) Circular genome maps of phages Brutus and Scipio. Circles from the inside outwards; circles with numbers inside; position in the genome; black: % GC content plotted around the average 37.40% (Brutus) and 37.60% (Scipio); olive/purple: GC skew. (**C**) Genetic map of phages Brutus and Scipio. Arrows indicate the direction of transcription. The scale bar indicates the length of the nucleotide sequence. Gene functions are shown in labels and legends.

*Acinetobacter* phages Brutus and Scipio have double-stranded DNA genomes of 44,931 and 44,602 base pairs (bp), respectively. The GC content of the genomes is 37.40% for Brutus and 37.60% for Scipio (Figure 2), which are close to typical values of *A. baumannii* strains (38–40%), probably indicating intensive horizontal gene exchange with host and/or a long history of host–parasite relations. The Brutus genome contains 91 predicted open-reading frames (ORFs) and the Scipio genome encodes 84 ORFs. No tRNA genes were found in the genomes. Consistent with the GC skew, counting from the 5′ end of the terminase gene module, the genomes include large blocks of packaging, virion and lysis genes oriented in the forward direction, blocks of about a dozen genes, including DNA recombination protein and DNA exonuclease, oriented in the reverse direction, and blocks of about 30 genes, including the replication genes, oriented in the forward direction again. The coding density of Brutus is 93.9% and that of Scipio is 92.7%. Codon usage is strongly biased toward A/T rich codons and the amino acids leucin (8.1% Brutus, 8.0% Scipio), lysine (7.8% Brutus, 7.6% Scipio), and isoleucine (7.3% Brutus, 6.9% Scipio), which are preferentially coded by A/T rich codons or A/T triplets only. Unlike other genes, the predicted genes of replication protein located upstream of DNA helicase contain short, direct, and repetitive sequences of nine to about twenty bp long in the middle of replication protein genes, suggesting the possible location of the origin of replication in the central part of the predicted genes of the replication protein, reminiscent of *Escherichia* phage λ [14], T4 [15], and plasmids [16]. According to the results of functional annotations (Section 4.5), including BLAST and HHpred searches, 56% of Brutus and 49% of Scipio predicted proteins did not show essential similarity to functionally characterized proteins and have been annotated as hypothetical proteins.

### 2.3. Search for Related Phages

To find all closely related phage genomes, genomic sequences of Brutus and Scipio and 19,460 *Caudoviricetes* phage sequences contained in the NCBI Genome database were used for calculations of the average nucleotide identity (ANI) (Supplementary Table S1). A sequence search employing amino acid sequences of major capsid proteins (MCPs) and a large subunit of terminase (TLS) on the NCBI nr/nt databases was used to find possible unidentified phage genomes. The searches revealed thirty closely related genomes: twenty-eight genomes (including Brutus and Scipio) characterized as *Acinetobacter* phages and mainly classified as phages of the genus *Obolenskvirus*, one genome labeled *Myoviridae* sp. isolate ct6H824, and one presumed to be a phage genome (assembly HumMal, Ma_F259_Contig_623), which was found among human gut metagenomic sequences (Table 1). In addition, several *Klebsiella* phages and metagenomic phage sequences demonstrated some degree of similarity to MCP or TLS sequences but did not show a significant degree of ANI, as was seen with the 30 genomes mentioned above. Eight *Acinetobacter* phages, labeled *Obolenskvirus* by the NCBI Taxonomy classification, were classified as *Obolenskvirus* phages by the International Committee in Taxonomy of Viruses (ICTV).

**Figure 3:**
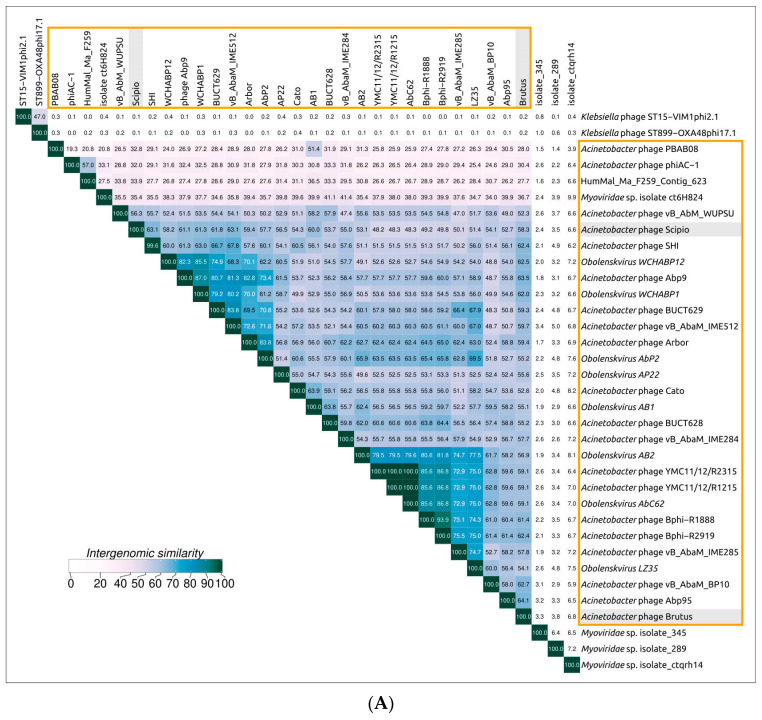

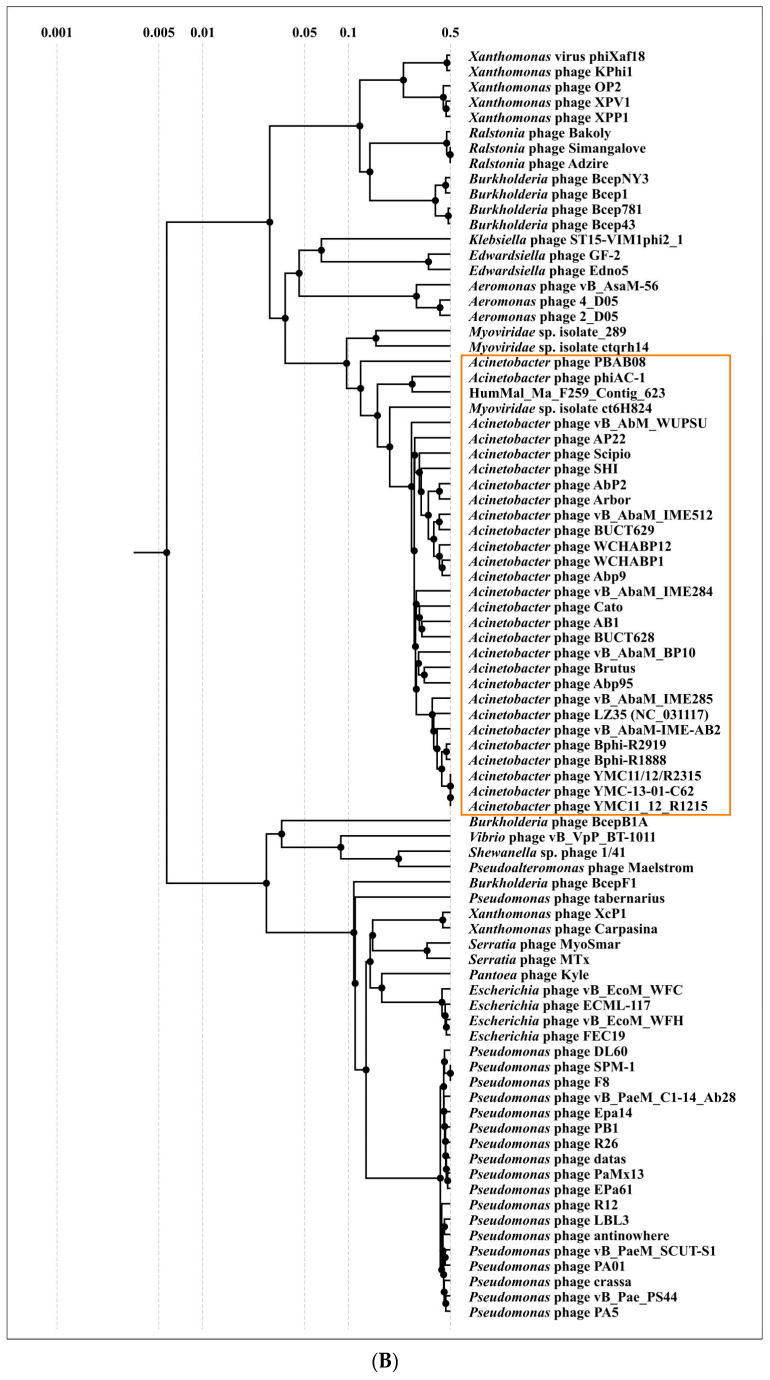
(**A**) Clustered heatmap based on the intergenomic similarity of phages Brutus and Scipio and related phages found with ANI calculations and a BLASTP search. The original VIRIDIC heatmap is shown in Figure S1. (**B**) Fragment of the “proteomic tree” inferred using the ViPtree server. A larger fragment of the original ViPtree using all reference genomes is shown in Figure S2. The group of phages referred to as “*Obolenskvirus*–like phages” is outlined in orange.

Clusterization performed with VIRIDIC, the tool recommended by the ICTV for intergenomic comparison and taxonomic assignment, confirmed the closeness of 30 of the genomes mentioned above (Figure 3A). Phylogenomic analysis carried out using ViP proteomic (Figure 3B) indicated that phages Brutus and Scipio belong to two distinct groups of *Acinetobacter* phages, but the VIRIDIC calculations showed difficulties in consistent clustering, indicating that some phages possess a high level of intergenomic similarity to phages of different VIRIDIC clusters and ViP proteomic branches. This can be a consequence of intensive genetic exchanges. In the following discussion, the group of 30 phages listed in Table 1 constituting a large cluster with intergenomic similarity of 19.3% and above in the VIRIDIC clustered heatmap (Figure 3A) and a large clade in the ViP proteomic tree (Figure 3B) will be referred to as the “*Obolenskvirus*–like group of phages” (OLG phages).

**Figure 4:**
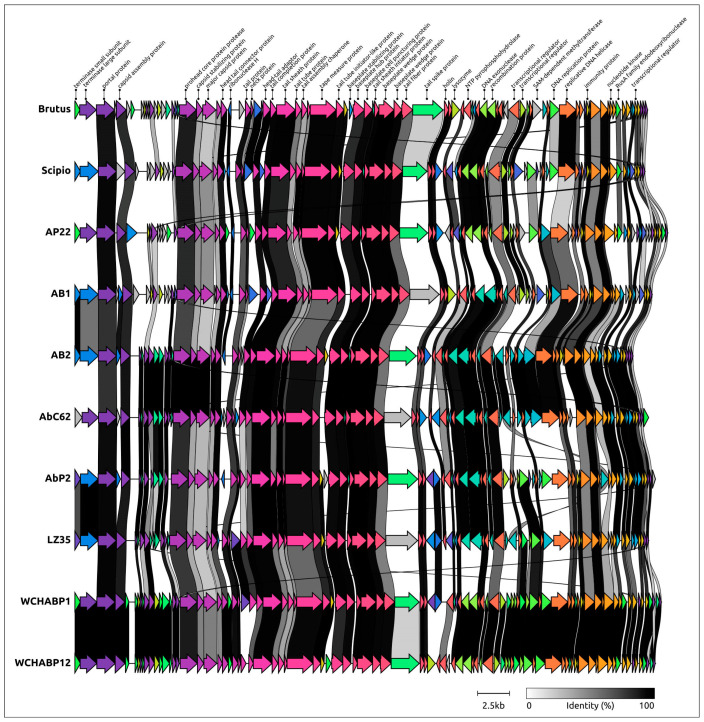
Genetic maps of ten *Obolenskvirus*-like phages comparing phage coding sequences (CDSs). Homologous proteins are assigned a unique colour and are linked by gray lanes showing the percentage of amino acid identity. The identity is represented by the intensity of gray, with darker shades of gray indicating a higher level of identity, according to the scale bar. Gene functions are shown in labels. Arrows indicate the direction of transcription. The scale bar 2.5 kb shows the genomic size.

All OLG phages are characterized by a similar organization of genomes (Figure 4), a genome size of 42–46 kbp, and a moderately low GC content of 37–39%. Almost all OLG genomes encode about 80–90 ORFs; the only exception is an unverified and unannotated genomic sequence labeled “*Acinetobacter* phage PBAB08”, in which a Glimmer prediction found 110 ORFs, most of which were shorter than in other OLG phage genomes. *Acinetobacter* phage phiAC-1 infects *Acinetobacter soli* strain KZ-1 and other isolated published phages infect *A. baumannii* strains (references are presented in Table 1). All of these phages demonstrated lytic activity, and no signs of the ability to lysogenize the host were shown, although *Obolenskvirus* AbP2 (*Acinetobacter* phage ϕAbP2) was originally described as a member of the subfamily *Peduovirinae*, mainly on the basis of limited phylogenetic analysis.

### 2.4. Functional Genome Content

Genomes of the phages Brutus and Scipio contain four distinct blocks of genes, assigned according to their functions to structural (morphogenetical), replication, lysis, and packaging modules. In turn, the morphogenetic module consists of two distinguishable parts of capsid and tail genes (Figure 2). This kind of genome organization, as well as the list of genes contained, is common for many different *Caudoviricetes* phages, including the *Obolenskvirus* phages.

**Figure 5:**
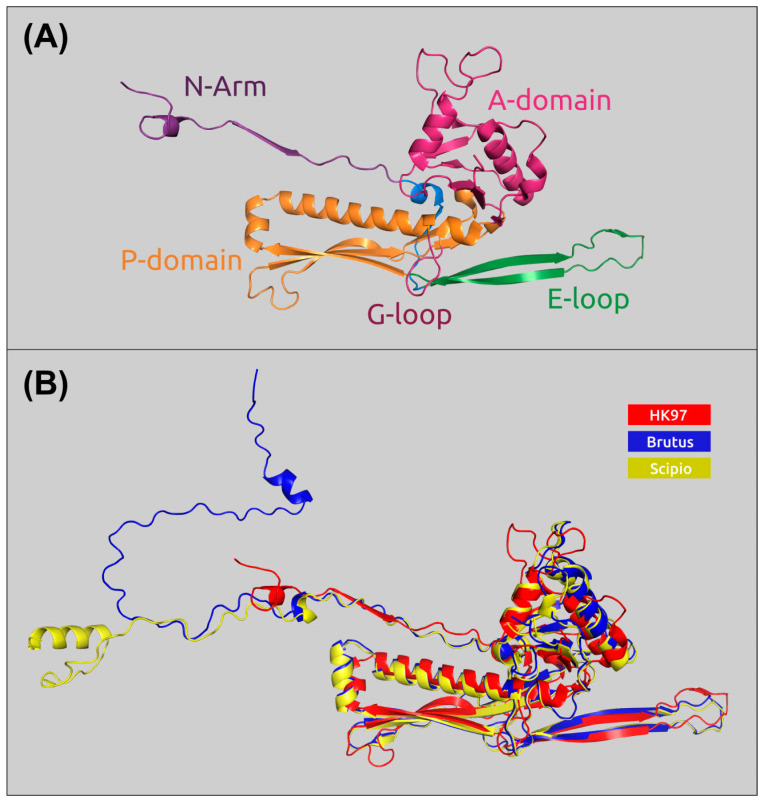
(**A**) A diagram of major capsid protein (MCP) in the mature HK97 capsid (PDB ID: 1OHG) and its common features, colored as indicated in the figure (according to Duda et al. [34]). (**B**) AlphaFold 2 models of MCPs of the phages Brutus and Scipio, superimposed onto the HK97 MCP.

AlphaFold2 (AlphaFold, AF2) [31] modeling showed the HK97-like major capsid proteins of Brutus and Scipio, featuring all the *Duplodnaviriaviruses* (Figure 5A). MCPs of these two phages are identical only in 34.6% of amino acid residues but possess a very similar structure; the structural alignment of AF2 causes an RMSD (root-mean-square deviation) of 0.697 Å. The scaffolding protein (SP), removed in the mature capsid of *Caudoviricetes* viruses, is encoded by the 5′ part of the MCP gene; the length of the SP is about 40–50 amino acids. Some bacteriophages contain MCPs with a more complicated structural architecture than phage HK97 and include additional domains, e.g., phage ϕ29 [32] and *Crassvirales* phages [33]. Analysis of *Obolenskvirus* MCPs indicated that they all had no such domains. Interestingly, the gene located upstream of the MCP gene encodes the capsid stabilizing protein. AF2 modeling and HHpred comparisons pointed to the similarity of this protein to this ϕ29 capsid fiber protein and other phage capsid proteins also named minor capsid and decoration proteins.

The packaging genomic modules of Brutus and Scipio contain genes of small and large terminase subunits (TSS and TLS). The TLS of phage Brutus (433 aa) is very similar to the TLS of phage AP22 (ab. 99% amino acid identity, 433 aa) but shares only 20% identity with the TLS of phage Scipio (474 aa). An HHpred search showed the closer proximity of the TLS nuclease domains of Brutus to the Pfam family PF17288 (Terminase_3) and that of phage Scipio to the Pfam family PF17289 (Terminase_6).

The lysis block of Brutus, Scipio, and other OLG phage genomes does not contain annotations for all members of the holin/lysozyme (also known as endolysin)/spanin lysis system, typically participating in *Caudoviricetes* viral particle release [35,36]. While the holin and endolysin genes can be easily found by homology and HMM search, the spanin component has not been predicted in any of the *Obolenskvirus* genomes. The existence of an orphan gene, located between the holin end lysozyme genes and encoding a transmembrane domain-containing protein, however, suggests that it may function as a spanin. Additionally, there are additional possible ORFs hypothetically involving alternate start codons. It appears to be difficult, now, to characterize the lysis module bioinformatically.

The tail module accounts for about a third of the Brutus and Scipio genomes. They are very similar in their gene content and contain 17 (Brutus) and 16 (Scipio) genes with predicted functions encoding structural and morphogenetic proteins and several orphan genes. The organization of the tail module is typical of myoviruses, including the presence of the tail sheath protein, and this is shared by all members of the *Obolenskvirus* group. According to AlphaFold predictions, the tail sheath proteins of Brutus and Scipio, which often have a multidomain structure, contain two domains in addition to the conserved part [37]. This structural architecture appears to be preserved for all *Obolenskvirus-*like phages.

Another third of Brutus, Scipio, and other OLG phage genomes contain different genes that are presumably involved in various processes, including replication and nucleic acid manipulations. The replication apparatus does not contain phage-encoded DNA polymerase but includes a predicted origin-specific replication protein, which is presumably responsible for the initiation of replication and DNA helicase, which is involved in replication. As with other phages [38], the nucleotide sequence of the replication protein contains repeats that may be related to the replication origin. Interestingly, Brutus and Scipio genomes encode S-adenosyl-methionine-dependent methyltransferase, which is likely to be involved in the protection of phage DNA from bacterial phage resistance mechanisms, as well as a putative immune protein that prevents superinfection of the infected cell.

### 2.5. Genome Architecture and Pangenome Analysis

**Figure 6:**
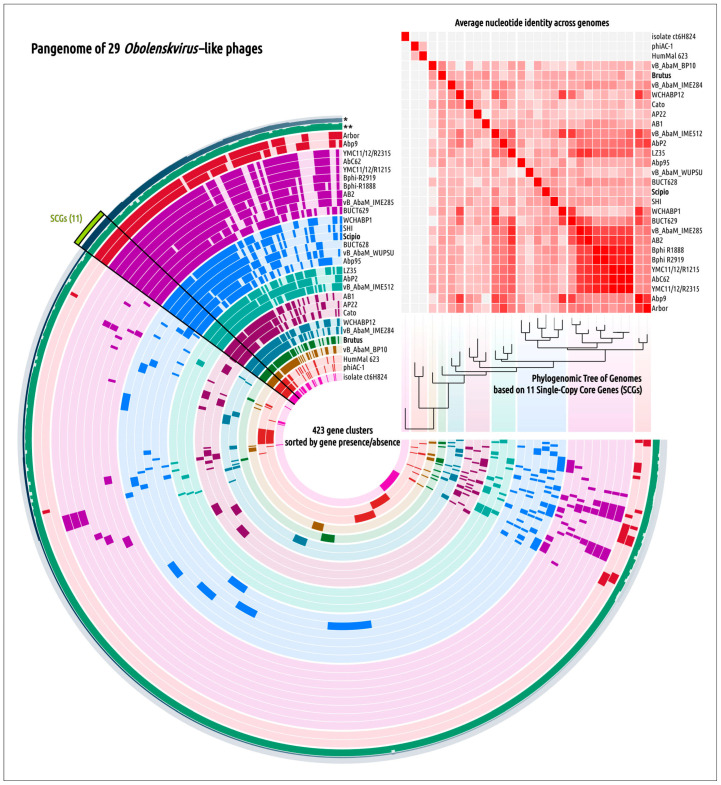
Pangenome analysis of the *Obolenskvirus*-like group of phages using 29 phage genomes. Genomes are organized according to their position in the phylogenomic tree based on 11 single-copy genes (SCGs). Gene clusters are shown as segments of radial layers, where the gene clusters present in genomes are colored in intense colors and the clusters absent in genomes are colored in pale shades. The more intense colors on the ANI heatmap indicate higher ANI values. The radial layer marked with a * shows the number of genes in the gene clusters; the width of the radial layer marked with ** represents the functional homogeneity index. HumMal 623 is an abbreviation of assembly HumMal Ma_F259_Contig_623; isolate ct6H824 is an abbreviation of *Myoviridae* sp. isolate ct6H824.

Pangenomic analysis of OLG phages revealed 11 single-copy genes (SCGs) that were shared by all the group members used to construct phylogeny based on the concatenated sequences (Figure 6). The list of these proteins included baseplate hub proteins, baseplate stabilizing proteins, baseplate wedge proteins, capsid assembly proteins, head–tail connectors, hypothetical proteins (Brutus_gp83), tail proteins (Brutus_gp27), tail sheath initiator proteins, tail sheath proteins, tail tube initiators, and tail tube proteins. However, the low level of similarity of such a conserved protein as MCP in evolution-related phages, which are close in lifestyle and genome organization, raises questions about the commonality of recent evolutionary history and the origin of some OLG proteins, as well as the appropriateness of using an approach that includes the concatenation of sequences of individual marker genes and proteins encoded by them for the purpose of evolutionary analysis.

**Figure 7:**
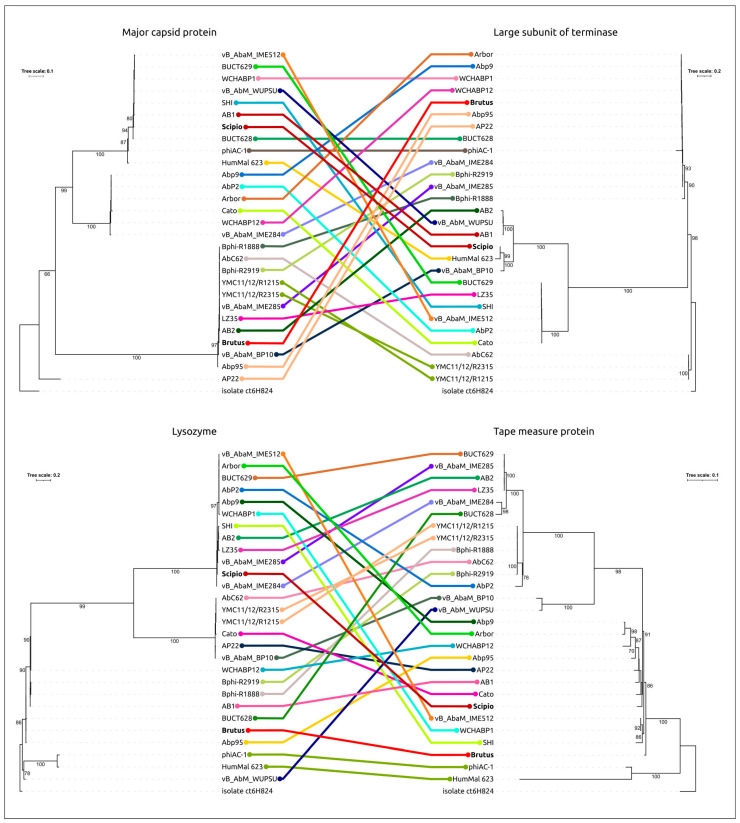
Best-scoring phylogenetic trees based on amino acid sequences of MCP, TLS, lysozyme, and TMP. HumMal 623 is an abbreviation of assembly HumMal Ma_F259_Contig_623 and isolate ct6H824 is an abbreviation of *Myoviridae* sp. isolate ct6H824. The trees were rooted to *Myoviridae* sp. isolate ct6H824. Identical phages are indicated with colored lines.

Phylogenetic analysis using sequences of major capsid protein, large subunits of terminase, lysozyme (endolysin), and tape measure proteins (TMPs) showed inconsistencies in the topologies of these phylogenies (Figure 7) concerning the order and composition of clades. Phages belonging to the same clade of one tree are often placed in different clades of another tree. In three trees, phages Brutus and Scipio belong to different clusters (MCP, TLS, lysozyme) but are grouped closely in the TMP tree. According to genetic distances, most clades contain very similar sequences but have different phages for different trees. This may indicate fast changes in sequences, which can be related to horizontal gene transfer (HGT) between the phages. The distribution of gene clusters, which is quite variable within the clades of the SCG-based tree, can also indicate intense gene transfers, as well as the complex origin of OLG genes.

**Figure 8:**
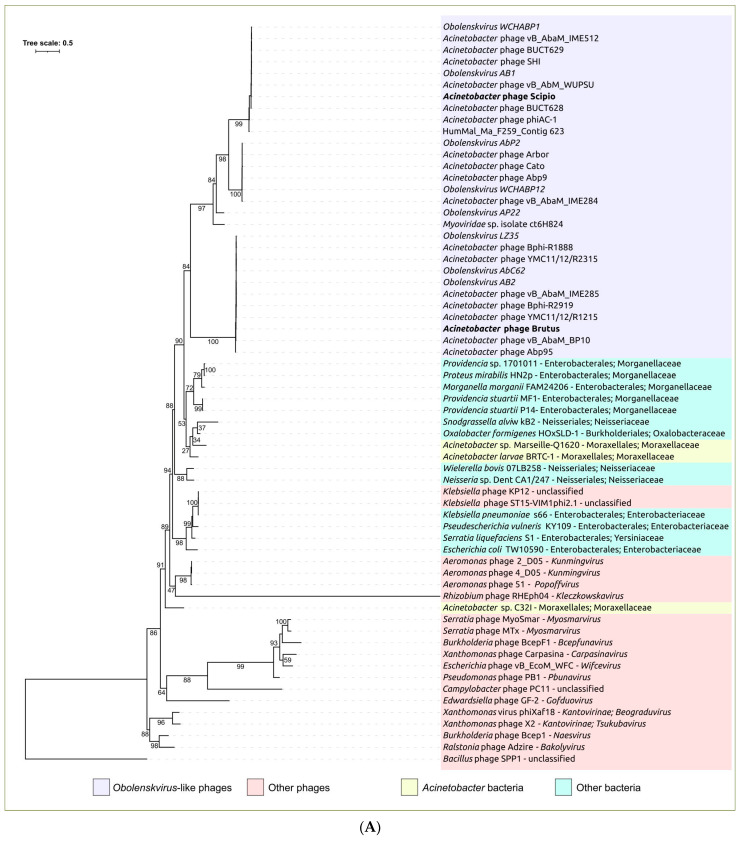

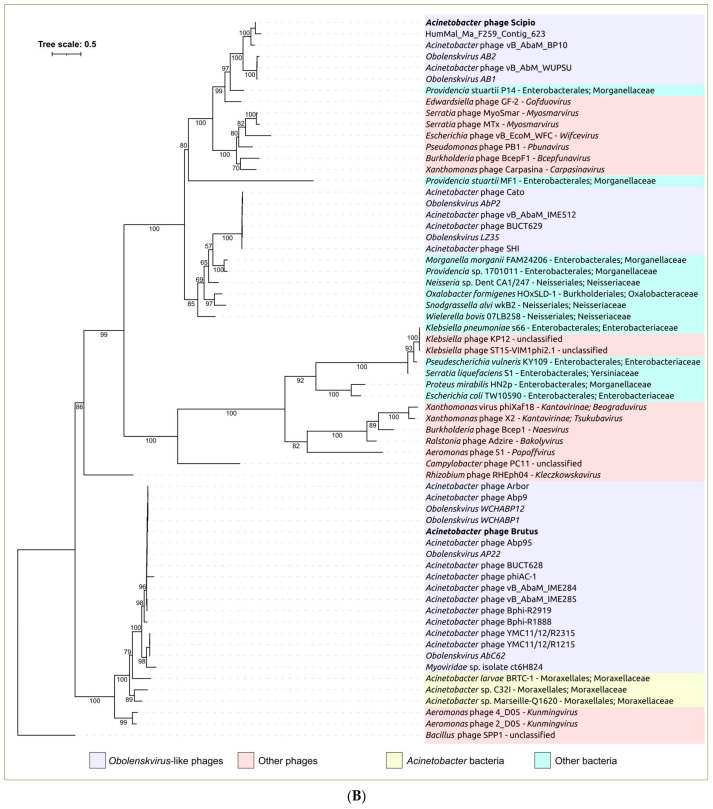
(**A**) Best-scoring ML phylogenetic tree constructed using amino acid sequences of major capsid proteins found in phage and bacterial genomes using Brutus and Scipio MCPs. (**B**) Best-scoring ML phylogenetic tree constructed using amino acid sequences of the large subunit of terminase found in phage and bacterial genomes using Brutus and Scipio TLSs. *Bacillus* phage SPP1 was used as an outgroup. Taxonomy is shown to the right of the organism’s (phage’s) name. The numbers near the tree branches indicate the TBE values. The total number of bootstrap trees was 1000. The scale bar shows 0.5 estimated substitutions per site.

To reveal the homologs of the *Obolenskvirus*-like group of phages MCPs and TLSs, a BLAST search using NCBI nr/nt and the GenBank Phage database was conducted (E-value < 10^−5^). The results of the search indicated the absence of homologs of these proteins in sequenced genomes of *Acinetobacter baumannii*. The closest homologs of the MCP and TLS of OLG phages were found in the genomes of *Acinetobacter larvae* strain BRTC-1 and some unclassified *Acinetobacter* strains. The MCP and TLS genes in those genomes belong to prophage regions. The phylogenetic analysis conducted using sequences from different viral and bacterial taxa, representing the closest groups of homologous sequences found with BLAST, showed that all MCP OLG sequences share a monophyletic branch close to two *Acinetobacter* bacterial prophage MCPs (Figure 8A). Unlike the MCP tree, the TLS phylogeny places OLS sequences in a polyphyletic group (Figure 8B). The branch, containing most of the OLG TLS sequences, is adjacent to the branch including *Acinetobacter* bacterial prophage TLS sequences, but the remaining part of *Obolenskvirus*-like group TLS sequences is positioned in two distinct clades neighboring bacterial non-*Acinetobacter* sequences and viral sequences from phages not infecting *Acinetobacter* strains.

The closest phages, found with a BLAST search using Brutus and Scipio MCP and TLS sequences, represent different classified and unclassified, presumably temperate, phages. The genomes of these phages encode integrase and other genes responsible for the temperate lifestyle and often share similarities with OLG phages in the context of genome organization. They are *Gofduovirus* phages (*Edwardsiella* phage GF-2 and phage Edno5), miscellaneous *Klebsiella* phages (GZ7, ST512-KPC3phi13.5, *Klebsiella* phage ST11-VIM1phi8.3, Mulock), the *Psychrobacter* phage pOW20-A, etc. Homologs of integrases encoded in the genomes of these phages were not found in OLG phage genomes. According to the HHpred search, several transcriptional regulators (Brutus gp63 and gp64 and Scipio gp54, gp55, gp60) positioned in the module responsible for replication and DNA manipulation show a clear resemblance with phage repressors and antirepressors participating in the lysogeny decision. A thorough HHpred search, however, could not detect the OLG protein similar to known integrases. These results may indicate the loss of the integrase gene and the transition to a lytic lifestyle in the ancestors of *Obolenskvirus*-like phages. One might hypothesize that the temperate ancestors of *Obolenskvirus*-like phages changed their lifestyle after switching the host to *A. baumannii*, as suggested by the presence of related prophages in genomes of other *Acinetobacter* bacteria and the relatedness of OLG phages and conserved proteins to temperate phages infecting other Gammaproteobacteria and their proteins.

### 2.6. Receptor-Binding Proteins

The first step in phage-host interaction, adsorption, initiates the infection process and is the main determinant of phage host range [39,40]. This step is defined by a specific interaction between bacterial cell surface structures that act as receptors and phage receptor-binding proteins (RBPs), namely the tailspike and/or tail fiber proteins. In tailed capsular-specific *A. baumannii* phages, host specificity is usually related to tailspike proteins (TSPs) or structural depolymerases degrading capsular polysaccharides (CPSs) of a certain composition and structure [41,42,43,44,45,46]. Phage adsorption in Gram-negative bacteria can also engage O-antigen oligo/polysaccharides and other bacterial surface structures [47,48]. Although most RBPs are made of tail fiber proteins (TFPs) and TSPs composed of homotrimeric complexes, other proteins, such as tail needle and baseplate proteins, may also participate in the adsorption step [48,49,50].

The structural modules of the Brutus and Scipio genomes contain gene-encoding TSPs (Brutus_gp46 and Scipio_gp39) and TFPs (Brutus_gp45 and Scipio_gp38), indicating the complex adsorption apparatus of the phages. Analysis of Brutus and Scipio putative RBPs was conducted using sequence comparisons, HHpred, and DALI searches. The analysis indicated an average level of similarity (67% pairwise identity) of Brutus and Scipio TFPs, which contain a carbohydrate-binding domain (CBD domain). The CBD domains are located in the C-terminal parts of proteins, in regions corresponding to about 180–283 aa (Brutus) and 180–276 aa (Scipio). The significant difference in the amino acid composition of the CBD domains of the phages may be due to the different structures of their receptors.

The amino acid identity of Brutus and Scipio TSPs was as low as 31%. Both TFP and TSP sequences of the two phages are remarkably similar in the N-part of corresponding proteins. The N-termini of TFPs and TSPs are usually involved in binding to the phage particle; thus, they were quite conservative within the same taxonomic group. The receptor-binding (depolymerase) domains are located in the remaining part of proteins and are responsible for specific recognition of certain bacterial surface structures [51].

According to the HHpred searches, the enzymatic domains of Brutus and Scipio TSPs are located in the central part of the proteins, in regions of about 120–500 aa for Brutus and 130–450 aa for Scipio. Interestingly, the BLAST search using the GenBank Bacterial database found the closest homologs of the TSPs in several *A. baumannii* genomes, while close homologs of TFPs were found in the genomes of other representatives of the genus *Acinetobacter*. These findings might illustrate possible lateral transfers of RPB modules with the participation of bacterial hosts [52].

It is noteworthy that *Obolenskvirus* Brutus and previously described *Friunavirus* vB_AbaP_APK116 [43] are able to infect *A. baumannii* bacterial hosts, both belonging to the K116 capsular type. This means that TSPs encoded in the phage genomes can specifically recognize and degrade the CPS of the same K116 structure. TSP Brutus_gp46 is very similar to TSP APK116_gp43 at the amino acid level (the coverage obtained to an E-value of 0.0 was 88%, with an identity of 80.46%). Thus, it can be assumed that the mechanism of the enzymatic activities of these TSPs is most likely the same.

### 2.7. The Mechanism of Enzymatic Activity of TSP Scipio_gp39

Phage Scipio is the first bacterial virus encoding tailspike depolymerase specific to the K82 CPS of *A. baumannii* to be described. The structure of *A. baumannii* LUH5534, belonging to the K82 capsular type, was already established [12]. To elucidate the mechanism of the depolymerase action, the purified K82 CPS of *A. baumannii* LUH5534 was cleaved with recombinant protein Scipio_gp39.

The resulting products of cleavage were fractionated by gel permeation chromatography to isolate oligosaccharide 1 (OS1) and a small amount of intact polysaccharide. The structure of the oligosaccharide was established by one- and two-dimensional ^1^H and ^13^C NMR spectroscopy and was confirmed by high-resolution electrospray ionization mass spectrometry (HR ESI-MS). The ^13^C NMR spectrum of the OS1 contained signals of four anomeric atoms of three linked monosaccharides (β-D-Gal*p* (unit **B**), α-D-Glc*p*NAc (unit **C**), and β-D-Gal*p*4,6Pyr (unit **D**) at δ 95.6–106.1 and one monosaccharide at the reducing end Gal*p*NAc (unit **A**) at δ 92.6 and 96.5 (for α- and β-anomer, Aα, and Aβ, respectively) (Figure S3).

The positive ion HR ESI mass spectrum showed a [M + H]^+^ peak of C_31_H_50_O_23_ at *m*/*z* 819.2872 (calculated value 819.2872), a [M + NH_4_]^+^ peak at *m*/*z* 836.3136 (calculated value 836.3143), a [M + Na]^+^ peak at *m*/*z* 841.2692 (calculated value 841.2697), and a [M + K]^+^ peak at *m*/*z* 857.2427 (calculated value 857.2436) (Figure S4). 

**Figure 9:**
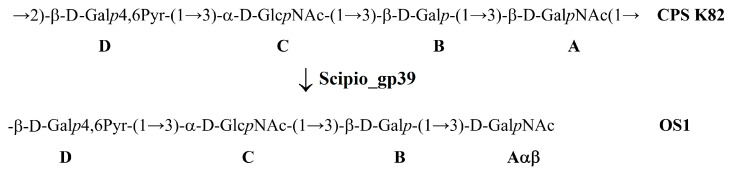
Cleavage of the K82 CPS of *A. baumannii* LUH5534 with Scipio_gp39 giving rise to oligosaccharide **1** (OS1) corresponding to the monomer of the K unit.

It can be concluded that oligosaccharide **1** was a tetrasaccharide, as shown in Figure 9.

The data obtained show that TSP Scipio_gp39 is a glycosidase that cleaves, specifically, the *A. baumannii* K82 CPS by the hydrolytic mechanism by the linkage between the repeating K units.

## 3. Discussion

In this work, the biological properties and genomic organization of two novel *Obolenskvirus* phages, Brutus and Scipio, were characterized. Phage Scipio is the first reported *A. baumannii* virus infecting the *A. baumannii* strain of the K82 capsular type. Thus, it was of particular interest to establish the mechanism of enzymatic activity of the tailspike depolymerase encoded in the phage genome. An analysis of the oligosaccharide product obtained by the degradation of the K82 CPS by the recombinant TSP Scipio_gp39 showed that the enzyme was a specific glycosidase cleaving the CPS by the hydrolytic mechanism to yield a monomer of the K repeating unit. Phage Brutus is capable of infecting the *A. baumannii* strain with a K116 CPS structure, like the previously described *Friunavirus* vB_AbaP_116 [43], which is confirmed by the similarity of TSPs Brutus_gp46 and APK116_gp43 at the amino acid level.

Comparative genomic, phylogenetic, and pangenomic analyses were performed to clarify the taxonomic position of the phages within OLG viruses and trace their possible origin and evolutionary history.

It is generally known that bacteriophages exhibit two distinct lifestyles: temperate and virulent [53]. Temperate phages are capable of entering a latent phase of infection within a host cell (lysogenic cycle), integrating their genome into the host chromosome (like phages λ [54] and P2 [55]) or existing as a circular (like phage P1 [56]) or a linear plasmid (like phage N15 [57]) in a bacterium. Virulent (lytic) phages (like T7 or T4 [58]) are characterized by a rapid infection accompanied by replication, progeny release, and bacterial lysis (lytic cycle). Temperate phages can also enter into the lytic cycle in certain circumstances, e.g., through being induced [59]. The lifestyle and genomics of phages are interconnected since the maintenance of lysogeny requires additional genes encoding components of the lysogenic apparatus, such as integrases (site-specific tyrosine recombinases), excisionases, and transcription regulators responsible for the lysogeny decision [60]. Temperate phages can be more prone to genetic exchanges [53] that can be related to recombinases encoded in their genomes [61]. Extensive gene exchange results in genetic mosaicism, and this is especially pronounced for temperate phages [53,54,62,63]. Genetic mosaicism manifests itself by a different evolutionary history of different genes and genetic modules, which can be seen from the different topologies of corresponding phylogenetic trees.

Phages Brutus and Scipio, like other *Obolenskvirus* phages, exhibit a strictly virulent lifestyle, and their genomes seem to not contain integrase or excisionase. The results of phylogenetic analyses performed in this study, however, indicate relationships between OLG phages and temperate phages that infect representatives of the genus *Acinetobacter*. These results enable speculation as to the origin of OLG phages from temperate phages infecting bacteria other than *A. baumannii*, and the loss of the ability to lysogenize the host might accompany host switching. The hypothesis of the origin of *Obolenskvirus* phages from temperate phages is supported by other indirect evidence. Like lambdoid phages, OLG phages do not encode their own DNA polymerase, seeming to use the host’s DNA polymerase. Genomes of *Obolenskviruses* encode a putative immunity protein, which supposedly participates in superinfection exclusion. This type of protein is especially useful for temperate phages [64]. The phylogenetic analysis of key proteins indicated a pervasive genetic mosaicism accompanying the evolution of OLG phages. The latest observations do not only apply to temperate phages but they are especially pronounced in this type of phage.

Phylogenetic analysis using sequences of major capsid protein and a large subunit of terminase showed the different evolutionary history of these proteins but, in both trees, the phages Brutus and Scipio were placed in different clusters. In the MCP tree, OLG phages form a monophyletic branch that diverges from the common ancestor of OLG phages and prophages of Gammaproteobacteria. This branch includes two distinct clusters, “Brutus-like” and “Scipio-like” phages. In the TLS tree, OLG phages are polyphyletic and are interspersed with various temperate phages. The incontinence of phylogenies of key proteins raises the previously discussed question of the correspondence between whole-proteome phylogeny and the true evolutionary history of phages and of the correspondences between taxonomy based on whole-proteome phylogeny and this history. The classification of OLG phages, including Brutus and Scipio, is also hampered by the results of calculations of intergenomic similarity, which mostly fail to achieve the common 70% genus threshold. To avoid the potential for spurious delineation of new taxa containing related and biologically similar phages, it would appear appropriate to lower the genus threshold and assign phages Brutus and Scipio to the genus *Obolenskvirus*, together with other OLG-like phages analyzed in this study.

## 4. Materials and Methods

### 4.1. Phage Isolation, Propagation, and Purification

Bacteriophages Brutus and Scipio were isolated from sewage samples collected in the Moscow region, in 2018, from bacterial lawns of *A. baumannii* strains MAR15-3273 and LUH5534, belonging to K116 and K82 capsular types, respectively. The samples were cleared by low-speed centrifugation (7000× *g* for 30 min), the supernatants were supplemented with LB medium and then incubated for 16–18 h, in the presence of growing *A. baumannii* strains belonging to different capsular types, at 37 °C, with shaking, and then chloroform was added. Bacterial debris was pelleted by centrifugation at 7000× *g* for 30 min, followed by filtration of the supernatants through 0.45 µm pore membrane filters (Merck Millipore, Cork, Ireland); the purified filtrates were then concentrated by ultracentrifugation at 85,000× *g* (Beckman SW50.1 Ti rotor, Beckman Coulter Inc., Brea, CA, 530 USA) at 4 °C for 2 h. The spot test method, as well as the plaque assay [65], was used to screen for the presence of lytic phage activity in the resulting concentrated preparations. The plates were incubated overnight at 37 °C and examined for zones of lysis or plaque formation.

Single plaques formed on the lawns of sensitive *A. baumannii* strains LUH5534 and MAR15-3273 were identified in the SM buffer (10 mM Tris-HCl, pH 7.5, 10 mM MgSO_4_, and 100 mM NaCl) and were replated three times in order to obtain pure phage stock. 

The phages Brutus and Scipio were propagated using a liquid culture of *A. baumannii* host strains MAR15-3273 and LUH5534 (OD_600_ of 0.3), respectively, at a multiplicity of infection (MOI) of 0.1. 

Phage particles were precipitated by PEG 8000 (at a final concentration of 10% and 500 mM NaCl). The final purification was executed by cesium chloride density-gradient centrifugation at 100,000× *g* (Beckman SW50.1 Ti rotor, Beckman Coulter Inc., Brea, CA, USA) for 24 h [66].

### 4.2. Phage Adsorption and One-Step Growth Experiments

For an adsorption assay, exponentially grown *A. baumannii* host cells LUH5534 and MAR15-3273 were mixed with the phages Scipio and Brutus (MOI = 0.001), respectively, and incubated at room temperature. A volume of 100 µL of the samples was taken after 0, 1, 3, 5, 8, 10, and 15 min, and then mixed with 850 µL of SM buffer supplemented with 50 µL of chloroform. After centrifugation, the supernatants were titrated for further determination of unabsorbed phages using the plaque assay method [65] at different time intervals. The adsorption constant was calculated according to a study by Adams [65] for a period of 5 min.

For the one-step growth experiments, 20 mL of host bacterial cells (OD_600_ ~ 0.3) was harvested by centrifugation (7000× *g* for 10 min at 4 °C) and resuspended in 1 mL LB broth. Bacterial cells were infected with the phages at an MOI of 0.01. The bacteriophages were allowed to adsorb for 5 min at 37 °C. Then, the mixtures were centrifuged at 10,000× *g* for 3 min to remove unabsorbed phage particles, and the pellets were resuspended in 20 mL of LB broth. Samples were taken at 5 and 10 min intervals during 1 h of incubation at 37 °C and immediately titrated.

The procedures were repeated three times.

### 4.3. Electron Microscopy

For negative staining, specimens were placed onto grids coated with formvar film and then, after drying, treated with a 0.3% aqueous solution of uranyl acetate (pH 4.0). The specimen samples were examined with a JEM-1400 (JEOL, Tokyo, Japan) transmission electron microscope at an accelerating voltage of 80 kV.

### 4.4. Phage DNA Isolation and Sequencing

Phage DNA was obtained from concentrated and purified high titer phage stock using a standard phenol-chloroform method [66], with previous incubation of the sample in 0.5% SDS and 50 µg/mL proteinase K at 65 °C for 20 min. The MiSeq platform and a Nextera DNA library preparation kit (Illumina, San Diego, CA, USA) were used for phage genome sequencing. The reads were prepared for assembly by Q15 filtering, quality trimming at the end of the reads, the removal of library adapter sequences, the removal of A/T stretches, and the exclusion of short reads (less than 60 bp). Raw sequence reads were deposited under BioProject PRJNA1070562. The generated reads were assembled *de novo* into a single contig using SPAdes v. 3.12 [67] with default parameters.

### 4.5. Phage Genome Annotation and Analysis

Open reading frames (ORFs) were predicted using Prodigal 2.6.1 [68], GeneMarkS 4.3 [69], and Glimmer 3.02 [70], and curated manually to ensure fidelity. Gene functions were assigned to ORFs using a BLAST [71] search in the NCBI databases (http://ncbi.nlm.nih.gov, accessed 17 May 2023) and custom phage databases InterPro [72] and HHpred [73], with a similarity criterion of HHpred probability of 95% and higher, using databases PDBmmCIF70, SCOPe70, ECOD, Pfam-A, and UniProt_Swiss_Prot_viral70. The functional assignment of some proteins was performed using structural prediction with AlphaFold 2.2 [31], and subsequent structural comparison was achieved using the DALI server [74]. tRNA coding regions were checked with ARAGORN [75]. The resulting genomes were visualized using Geneious Prime, version 2023.1 (Biomatters, Inc., Auckland, New Zealand). Annotated genomes of phages Brutus and Scipio have been deposited in the NCBI GenBank under accession numbers ON036882 and ON036883, respectively.

The genetic maps were visualized using Geneious Prime, CGView [76], and Clinker [77]. Phage genomes were downloaded from the NCBI GenBank database. Comparisons between the phage genomes were performed and visualized using Clinker, applying the default settings for the estimation of similarities among genomic loci. An average nucleotide identity search was conducted using orthoANI [78], with default settings, and the NCBI Genome database containing *Caudoviricetes* sequences. Pairwise nucleotide similarities among the phage genomes and the corresponding clustered heatmap were computed using orthoANIu and VIRIDIC [79], with default settings being used. Codon usage was estimated with Geneious Prime and GC skew and CG content were assessed and visualized with CGView. The phylogenomic tree was inferred using the Genome-BLAST Distance. Pangenome analysis was conducted using Anvi’o workflow and commands “anvi-gen-contigs-database”, “anvi-script-gen-genomes-file”, “anvi-gen-genomes-storage”, “anvi-pan-genome”, “anvi-display-pan”, “anvi-compute-genome-similarity”, “anvi-get-sequences-for-gene-clusters”, “trimal-gt 0.50”, and “iqtree-s-m WAG, -bb1000” [80].

### 4.6. Phylogenetic Analysis

Protein sequence alignments were obtained using MAFFT 7.48 with default settings, using the L-INS-i algorithm [81]. The terminase phylogenetic tree was constructed using the RAxML-NG 1.1.0 [82] built-in raxmlGUI 2.0.10 graphical user interface [83], applying “--bs-metric tbe--tree rand{10}--bs-trees 1000” settings. The best amino acid substitution models were estimated using ModelTest-NG [84]. The robustness of the tree was assessed using a bootstrap analysis employing 10 starting trees and 1000 bootstrap replicants before calculating the transfer bootstrap expectation (TBE) values. The trees were visualized using the iTOL server [85].

### 4.7. Protein Analysis and Structure Prediction

All monomer protein structures were modeled using AlphaFold 2.2, with full databases and the command line parameter “-monomer”, and the best-ranked structures were selected for further analysis. Protein complexes were modeled using AlphaFold 2.2 with command line parameter “-multimer” [86]. Protein structures were superimposed and visualized using Pymol 2.5.4 (Schrödinger Inc., New York, NY, USA). The robustness of structural alignments was assessed using root-mean-square deviation (RMSD), calculated using Pymol. Structure comparison was performed using the DALI server, with default settings. Structural similarity was evaluated with the DALI Z-score, and a structural similarity matrix was obtained using the DALI server. Phylogenetic trees based on structural similarity were obtained using the built-in DALI tools. The functional assignment of protein domains was performed through BLASTP, HHpred, and DALI searches.

### 4.8. Cloning, Expression, and Purification of the Recombinant TSP

The phage DNA sequence corresponding to the TSP Scipio_gp39 lacking N-terminal domain was amplified using PCR with the primers 5′-ataggatccaacaacggcatgtcttatgag-3′ and 5′-ataaagcttaaactattgcagaagtagcac-3′ and then cloned into pTSL plasmid [87]. Expression vectors were transformed into chemically competent *Escherichia coli* B834 (DE3) cells. Protein expression was performed in an LB medium supplemented with ampicillin at 100 µg/mL. Transformed cells were grown at 37 °C until the optical density reached a value of 0.6 at 600 nm. The medium was cooled to a temperature of 18 °C, followed by expression induction by the addition of isopro-pyl-1-thio-β-d-galactopyranoside (IPTG) to a final concentration of 1.0 mM before further incubation for 8 h at 18 °C. The cells were harvested by centrifugation at 4000× *g* for 20 min at 4 °C. Then, the cell pellet was resuspended in buffer A (20 mM Tris pH 8.0, 0.4 M NaCl) and sonicated (Virsonic, VirTis, NY, USA). The lysate was cleared by centrifugation at 13,000× *g* for 25 min and then loaded into 5 mL Ni^2+^-charged GE HisTrap columns (GE Healthcare Life Sciences, Chicago, IL, USA) equilibrated with buffer A. The proteins were eluted by a 0–200 mM imidazole step gradient in buffer A. His-tag and SlyD digestion was realized by incubation with tobacco etch virus (TEV) protease at a protease/protein ratio of 1/100 (*wt*/*wt*) overnight, with simultaneous dialysis against 10 mM Tris pH 8.0 containing 1.0 mM 2-mercaptoethanol. Each cleaved protein was loaded onto a 5 mL SourceQ 15 (GE Healthcare Life Sciences, Chicago, IL, USA) column and eluted with a linear gradient of 0–600 mM NaCl in 20 mM Tris-HCl (pH 8.0). Protein-containing fractions were combined and concentrated using Sartorius ultrafiltration devices (Sartorius AG, Gottingen, Germany), with a molecular weight cutoff of 50 kDa.

### 4.9. Isolation of the K82 Capsular Polysaccharide

The *A. baumannii* strain LUH5534 (K82) was cultivated in 2TY (16 g Bacto tryptone, 10 g Bacto yeast extract, 5 g NaCl) media overnight at 37 °C. Bacterial cells were harvested by centrifugation at 10,000× *g* for 20 min, washed with phosphate-buffered saline, suspended in aqueous 70% acetone, precipitated, and dried in air.

A sample of CPS (K82) was isolated using a phenol water extraction protocol [88]. Dried *A. baumannii* cells were incubated with 45% aqueous phenol for 60 min at 70 °C. The extract was cooled and dialyzed. Insoluble contaminations were removed by centrifugation at 12,000× *g* for 20 min. Aqueous 50% CCl_3_CO_2_H was added to a CPS solution in water at 4 °C; the precipitate was removed by centrifugation and the supernatant was dialyzed with distilled water and freeze-dried. CPS preparation was heated with 2% HOAc at 100 °C for 2 h. Then, a lipid precipitate was removed by centrifugation at 12,000× *g* for 20 min. Purified CPS samples were isolated from the supernatant by gel permeation chromatography on an XK 26 mm (width) × 70 cm (height) column (gel layer, 560 mm) (GE Healthcare Life Sciences, Chicago, IL, USA) of Sephadex G-50 Superfine (Amersham Biosciences, Uppsala, Sweden) in a 0.05 M pyridinium acetate buffer (pH 4.5).

### 4.10. Depolymerisation of the K82 CPS by Recombinant Tailspike Depolymerase 

Purified K82 CPS was solubilized in a 20 mM Tris-HCl pH 8.0 buffer, and 200–500 μg of recombinant TSP was added for digestion. The reaction mixture was incubated at 37 °C. CPS digestion products were fractionated by gel permeation chromatography on an XK 16 mm × 100 cm column (gel layer, 800 mm) (GE Healthcare Life Sciences, Chicago, IL, USA) of Fractogel TSK HW-40S (Toyo Soda, Tokyo, Japan) in 1% acetic acid.

### 4.11. NMR Spectroscopy

A sample of purified K82 CPS was deuterium exchanged and examined as a solution in 99.95% D_2_O on a Bruker Avance II 600 MHz spectrometer (Bruker Daltonics, Bremen, Germany). Sodium 3-trimethylsilylpropanoate-2,2,3,3-d_4_ (δ_H_ 0, δ_C_ −1.6) was used as an internal reference for calibration. Two-dimensional ^1^H-^1^H correlation spectroscopy (COSY), ^1^H-^1^H total correlation spectroscopy (TOCSY), ^1^H-^1^H rotating-frame nuclear Overhauser effect spectroscopy (ROESY), ^1^H-^13^C heteronuclear single-quantum coherence (HSQC), and ^1^H-^13^C heteronuclear multiple-bond correlation (HMBC) experiments were performed using standard Bruker software (Bruker TopSpin 3.6.0 program). The Bruker TopSpin 2.1 program was used to acquire and process the NMR data. A spin lock time of 60 ms and mixing time of 200 ms were used in ^1^H-^1^H TOCSY and ^1^H-^1^H ROESY experiments, respectively. A ^1^H-^13^C HMBC experiment was recorded, with a 60 ms delay for the evolution of long-range couplings, to optimize the spectrum for coupling constant J_H,C_ 8 Hz.

### 4.12. Mass Spectroscopy

High-resolution electrospray ionisation (HR ESI) mass spectrometry was performed in positive ion mode using a maXis instrument (Bruker Daltonics, Bremen, Germany). An oligosaccharide sample (~50 ng L^−1^) was dissolved in a 1:1 (*v*/*v*) water–acetonitrile mixture and injected, with a syringe, at a flow rate of 3 μL min^−1^. Capillary entrance voltage was set at –4500 V (positive ion mode) or 4000 V (negative ion mode). The interface temperature was set at 180 °C or 200 °C. Nitrogen was used as a drying and nebulizing gas. Mass spectra were acquired within the range of *m*/*z* 50 to *m*/*z* 3000. Internal or external calibration was carried out with ESI Calibrant Solution (Agilent, Santa Clara, CA, USA).

## Figures and Tables

**Table 1:** List of phages closely related to *Acinetobacter* phages Brutus and Scipio. Classified and approved by the ICTV (as of June 2023), *Obolenskvirus* phages (colored orange) are named according to the ICTV taxonomy.

#	Phage	NCBI Accession	Sequence Length, b.p.	% GC	ORF #	Reference
1	*Acinetobacter* phage Abp9	MN166083.1	44,820	37.70%	88	[17]
2	*Acinetobacter* phage Abp95	MZ618622.1	43,176	37.80%	84	[18]
3	*Acinetobacter* phage Arbor	ON237674.1	45,041	37.40%	85	
4	*Acinetobacter* phage Bphi-R1888	MN516422.1	44,590	37.90%	83	
5	*Acinetobacter* phage Bphi-R2919	MN516421.1	44,227	37.80%	82	
9	*Acinetobacter* phage Brutus	ON036882.1	44,931	37.40%	91	
7	*Acinetobacter* phage BUCT628	MZ593728.1	44,935	37.50%	86	[19]
8	*Acinetobacter* phage BUCT629	MZ712044.1	46,325	37.60%	88	[20]
9	*Acinetobacter* phage Cato	OM471864.1	45,188	37.40%	87	[21]
10	*Acinetobacter* phage PBAB08	MG366114.1	42,312	38.60%	110	[22]
11	*Acinetobacter* phage phiAC-1	NC_028995.1	43,216	38.50%	82	[23]
12	*Acinetobacter* phage Scipio	ON036883.1	44,602	37.60%	84	
13	*Acinetobacter* phage SHI	ON480525.1	44,180	37.60%	91	
14	*Acinetobacter* phage vB_AbaM_BP10	OP585104.1	44,443	37.30%	90	
15	*Acinetobacter* phage vB_AbaM_IME284	MH853787.1	43,557	38.30%	86	
16	*Acinetobacter* phage vB_AbaM_IME285	MH853786.1	45,063	37.90%	84	[24]
17	*Acinetobacter* phage vB_AbaM_IME512	MH853788.1	46,499	37.60%	91	
18	*Acinetobacter* phage vB_AbM_WUPSU	OL743187.1	44,241	37.20%	82	[25]
19	*Acinetobacter* phage YMC11/12/R1215	KP861231.1	44,866	37.60%	85	
20	*Acinetobacter* phage YMC11/12/R2315	NC_028855.1	44,846	37.60%	86	
21	Assembly HumMal, Ma_F259_Contig_623	CYGL01000085.1	45,317	37.80%	83	
22	*Myoviridae* sp. isolate ct6H824	BK017052.1	42,126	37.70%	85	
23	*Obolenskvirus* AB1	NC_042028.1	45,159	37.70%	85	[26]
24	*Obolenskvirus* AB2	NC_041857.1	43,665	37.50%	82	[26]
25	*Obolenskvirus* AbC62	NC_024785.1	44,844	37.60%	85	
26	*Obolenskvirus* AbP2	NC_041998.1	45,373	37.80%	87	[27]
27	*Obolenskvirus* AP22	NC_017984.1	46,387	37.70%	91	[28]
28	*Obolenskvirus* LZ35	NC_031117.1	44,885	37.90%	82	[29]
29	*Obolenskvirus* WCHABP1	NC_041966.1	45,888	37.60%	92	[30]
30	*Obolenskvirus* WCHABP12	NC_041924.1	45,415	37.60%	93	[30]

## Data Availability

Annotated genomes of *A. baumannii* phages Brutus and Scipio were deposited in GenBank under accession numbers ON036882 and ON036883, respectively. Raw sequence reads were deposited under BioProject PRJNA1070562.

## Supplementary Materials

The supporting information can be downloaded at https://www.mdpi.com/article/10.3390/ijms25042074/s1.
