# Supplementary material for: New Obolenskvirus Phages Brutus and Scipio: Biology, Evolution, and Phage-Host Interaction
Source: Int J Mol Sci. 2024 Feb 8;25(4):2074. doi: 10.3390/ijms25042074 (PMC10888812; doi:10.3390/ijms25042074)
Supplement: Supplementary file 1 [file ijms-25-02074-s001.zip › Supplementary materials_Figures_S3,S4_Table_S1.pdf]

**Figure S3.** The $^{13}$C NMR spectrum of the **oligosaccharide 1 (OS1)** obtained after cleavage of K82 CPS of *A. baumannii* LUH5534 with recombinant protein Scipio_gp39.

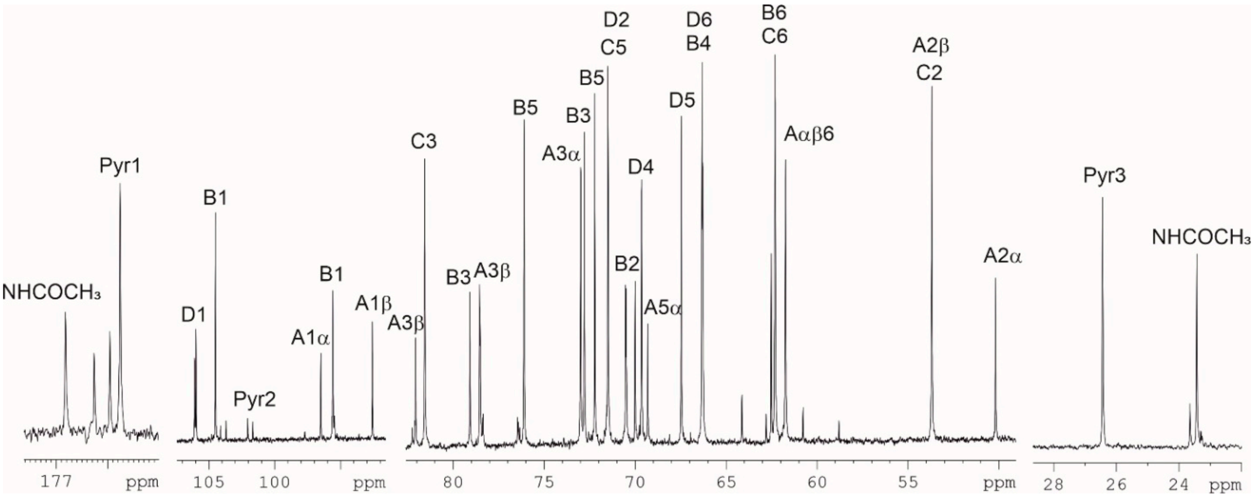


**Figure S4.** The positive ion mode HR ESI mass spectrum of the OS1.

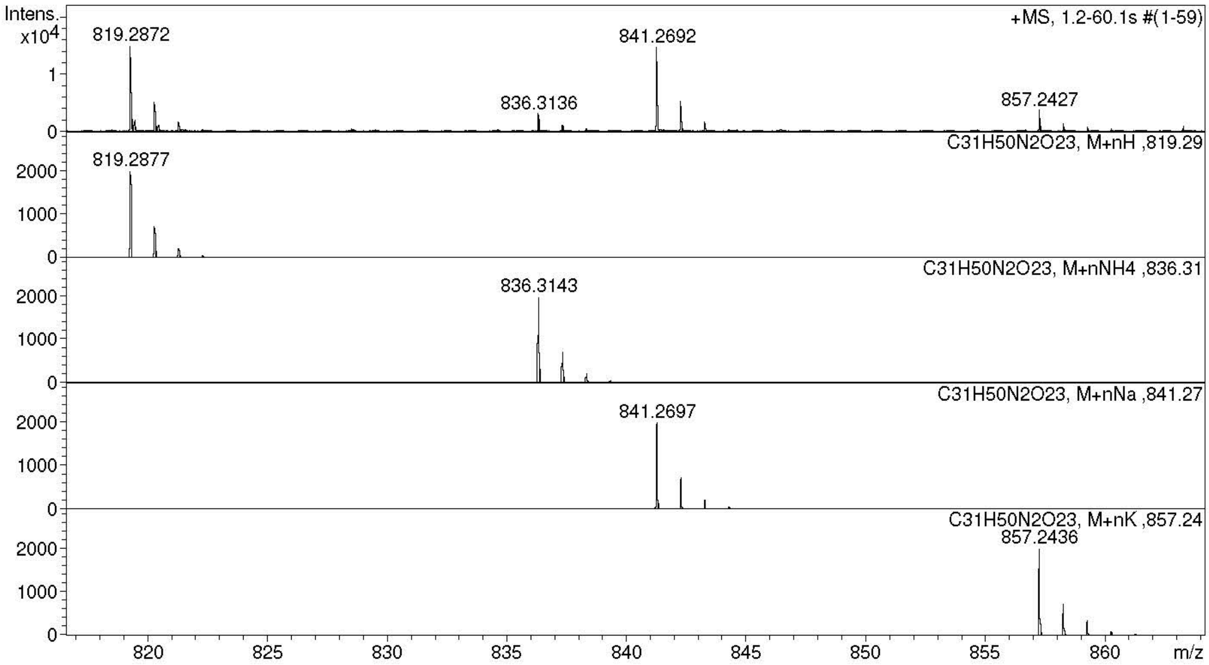

**Table S1.** Results of ANI calculations with orthoANIu using genomes of phages Brutus and Scipio compare to 19,460 *Caudoviricetes* phage sequences contained in the NCBI Genome. Cells with the coverage values of 0.16 and more is highlighted with red background.

| NCBI Accession | ANI | Aligned length, nt | Coverage genome 1 | Coverage genome 2 | Genome size, nt | NCBI Name | NCBI Taxonomy |
|---|---|---|---|---|---|---|---|
| phage Brutus | | | | | | | |
| MG366114 | 92.36 | 7076 | 0.1613 | 0.1692 | 41820 | *Acinetobacter* phage PBAB08 | *Myoviridae* |
| OP585104 | 92.35 | 13850 | 0.3158 | 0.3158 | 43860 | *Acinetobacter* phage vB_AbaM_BP10 | *Obolenskvirus* |
| MN516421 | 90.47 | 18032 | 0.4111 | 0.4111 | 43860 | *Acinetobacter* phage Bphi-R2919 | *Obolenskvirus* |
| MZ593728 | 90.34 | 16165 | 0.3686 | 0.3602 | 44880 | *Acinetobacter* phage BUCT628 | *Obolenskvirus* |
| MZ618622 | 90.24 | 21498 | 0.4902 | 0.5018 | 42840 | *Acinetobacter* phage Abp95 | *Obolenskvirus* |
| MN166083 | 90.22 | 19858 | 0.4528 | 0.4528 | 43860 | *Acinetobacter* phage Abp9 | *Obolenskvirus* |
| KY829116 | 89.96 | 18862 | 0.4301 | 0.4203 | 44880 | *Acinetobacter* phage WCHABP1 | *Obolenskvirus* |
| OM471864 | 89.86 | 14484 | 0.3302 | 0.3227 | 44880 | *Acinetobacter* phage Cato | *Obolenskvirus* |
| MN807295 | 89.70 | 700 | 0.0160 | 0.0172 | 40800 | *Acinetobacter* phage vB_AbaP_APK116 | *Autographiviridae* |
| HM368260 | 89.67 | 15024 | 0.3425 | 0.3348 | 44880 | *Acinetobacter* phage AB1 | *Obolenskvirus* |
| JX976549 | 89.62 | 17528 | 0.3996 | 0.4092 | 42840 | *Acinetobacter* phage vB_AbaM-IME-AB2 | *Obolenskvirus* |
| ON480525 | 89.42 | 18710 | 0.4266 | 0.4266 | 43860 | *Acinetobacter* phage SHI | *Obolenskvirus* |
| KJ817802 | 89.33 | 17000 | 0.3876 | 0.3876 | 43860 | *Acinetobacter* phage YMC-13-01-C62 | *Obolenskvirus* |
| MZ712044 | 89.17 | 17473 | 0.3984 | 0.3807 | 45900 | *Acinetobacter* phage BUCT629 | *Obolenskvirus* |
| ON237674 | 89.03 | 16122 | 0.3676 | 0.3592 | 44880 | *Acinetobacter* phage Arbor | *Obolenskvirus* |
| MN516422 | 88.95 | 18947 | 0.4320 | 0.4320 | 43860 | *Acinetobacter* phage Bphi-R1888 | *Obolenskvirus* |
| KP861231 | 88.70 | 18160 | 0.4140 | 0.4140 | 43860 | *Acinetobacter* phage YMC11/12/R1215 | *Obolenskvirus* |
| ON036883 | 88.68 | 15460 | 0.3525 | 0.3525 | 43860 | *Acinetobacter* phage Scipio | *Obolenskvirus* |
| MH853788 | 88.57 | 20282 | 0.4624 | 0.4419 | 45900 | *Acinetobacter* phage vB_AbaM_IME512 | *Obolenskvirus* |
| MF346584 | 88.54 | 18159 | 0.4140 | 0.4046 | 44880 | *Acinetobacter* phage AbP2 | *Obolenskvirus* |
| HE806280 | 88.29 | 14079 | 0.3210 | 0.3067 | 45900 | *Acinetobacter* phage AP22 | *Obolenskvirus* |
| KP861229 | 88.16 | 17225 | 0.3927 | 0.3927 | 43860 | *Acinetobacter* phage YMC11/12/R2315 | *Obolenskvirus* |
| KU510289 | 87.69 | 18851 | 0.4298 | 0.4200 | 44880 | *Acinetobacter* phage LZ35 | *Obolenskvirus* |
| MH853787 | 87.47 | 17005 | 0.3877 | 0.3969 | 42840 | *Acinetobacter* phage vB_AbaM_IME284 | *Obolenskvirus* |
| KY670595 | 87.39 | 23917 | 0.5453 | 0.5329 | 44880 | *Acinetobacter* phage WCHABP12 | *Obolenskvirus* |
| OL743187 | 87.17 | 16174 | 0.3688 | 0.3688 | 43860 | *Acinetobacter* phage vB_AbM_WUPSU | *Obolenskvirus* |
| MH853786 | 87.06 | 20188 | 0.4603 | 0.4498 | 44880 | *Acinetobacter* phage vB_AbaM_IME285 | *Obolenskvirus* |
| JX560521 | 72.88 | 8349 | 0.1904 | 0.1949 | 42840 | *Acinetobacter* phage phiAC-1 | *Myoviridae* |
| MN855951 | 69.80 | 567 | 0.0129 | 0.0695 | 8160 | *Siphoviridae* sp. | *Siphoviridae* |
| MN855957 | 66.85 | 1108 | 0.0253 | 0.0572 | 19380 | *Myoviridae* sp. | *Myoviridae* |
| KP137437 | 66.70 | 561 | 0.0128 | 0.0108 | 52020 | *Mannheimia* phage vB_MhS_1152AP2 | *Siphoviridae* |
| KP137435 | 65.70 | 561 | 0.0128 | 0.0117 | 47940 | *Mannheimia* phage vB_MhS_587AP2 | *Siphoviridae* |
| KP137433 | 65.30 | 561 | 0.0128 | 0.0112 | 49980 | *Mannheimia* phage vB_MhS_535AP2 | *Siphoviridae* |
| KP137439 | 65.30 | 561 | 0.0128 | 0.0108 | 52020 | *Mannheimia* phage vB_MhS_3927AP1 | *Siphoviridae* |
| MT664721 | 64.60 | 828 | 0.0189 | 0.0232 | 35700 | *Escherichia* phage vB_EcoM_APEC | *Podoviridae* |
| MN856055 | 64.35 | 1397 | 0.0319 | 0.0351 | 39780 | *Myoviridae* sp. | *Myoviridae* |
| KF669656 | 63.60 | 1342 | 0.0306 | 0.0337 | 39780 | *Acinetobacter* phage Petty | *Autographiviridae* |
| MN856093 | 60.60 | 720 | 0.0164 | 0.0543 | 13260 | *Myoviridae* sp. | *Myoviridae* |
| MN856091 | 60.40 | 674 | 0.0154 | 0.0245 | 27540 | *Myoviridae* sp. | *Myoviridae* |
| MT028491 | 59.70 | 653 | 0.0149 | 0.0029 | 227460 | *Ochrobactrum* phage vB_OspM_OC | *Myoviridae* |
| MN856109 | 56.50 | 762 | 0.0174 | 0.1067 | 7140 | *Myoviridae* sp. | *Myoviridae* |
| MN856004 | 54.00 | 756 | 0.0172 | 0.0176 | 42840 | *Myoviridae* sp. | *Myoviridae* |

| phage Scipio | | | | | | | |
|---|---|---|---|---|---|---|---|
| MZ712044 | 91.76 | 13421 | 0.3060 | 0.2924 | 45900 | *Acinetobacter* phage BUCT629 | *Obolenskvirus* |
| KU510289 | 89.95 | 13900 | 0.3169 | 0.3097 | 44880 | *Acinetobacter* phage LZ35 | *Obolenskvirus* |
| MH853788 | 89.78 | 20329 | 0.4635 | 0.4429 | 45900 | *Acinetobacter* phage vB_AbaM_IME512 | *Obolenskvirus* |
| ON480525 | 89.74 | 18529 | 0.4225 | 0.4225 | 43860 | *Acinetobacter* phage SHI | *Obolenskvirus* |
| JX976549 | 89.73 | 12816 | 0.2922 | 0.2992 | 42840 | *Acinetobacter* phage vB_AbaM-IME-AB2 | *Obolenskvirus* |
| KY670595 | 89.49 | 15520 | 0.3539 | 0.3458 | 44880 | *Acinetobacter* phage WCHABP12 | *Obolenskvirus* |
| ON036882 | 89.37 | 13748 | 0.3135 | 0.3063 | 44880 | *Acinetobacter* phage Brutus | *Obolenskvirus* |
| KP861229 | 89.33 | 13371 | 0.3049 | 0.3049 | 43860 | *Acinetobacter* phage YMC11/12/R2315 | *Obolenskvirus* |
| MN516422 | 89.29 | 11937 | 0.2722 | 0.2722 | 43860 | *Acinetobacter* phage Bphi-R1888 | *Obolenskvirus* |
| MN516421 | 89.18 | 12472 | 0.2844 | 0.2844 | 43860 | *Acinetobacter* phage Bphi-R2919 | *Obolenskvirus* |
| KP861231 | 89.18 | 10826 | 0.2468 | 0.2468 | 43860 | *Acinetobacter* phage YMC11/12/R1215 | *Obolenskvirus* |
| MF346584 | 88.78 | 14478 | 0.3301 | 0.3226 | 44880 | *Acinetobacter* phage AbP2 | *Obolenskvirus* |
| MH853787 | 88.66 | 14908 | 0.3399 | 0.3480 | 42840 | *Acinetobacter* phage vB_AbaM_IME284 | *Obolenskvirus* |
| KY829116 | 88.54 | 20295 | 0.4627 | 0.4522 | 44880 | *Acinetobacter* phage WCHABP1 | *Obolenskvirus* |
| MZ593728 | 88.43 | 14472 | 0.3300 | 0.3225 | 44880 | *Acinetobacter* phage BUCT628 | *Obolenskvirus* |
| OL743187 | 88.35 | 15110 | 0.3445 | 0.3445 | 43860 | *Acinetobacter* phage vB_AbM_WUPSU | *Obolenskvirus* |
| HM368260 | 87.69 | 17485 | 0.3987 | 0.3896 | 44880 | *Acinetobacter* phage AB1 | *Obolenskvirus* |
| OM471864 | 87.68 | 16067 | 0.3663 | 0.3580 | 44880 | *Acinetobacter* phage Cato | *Obolenskvirus* |
| MN166083 | 87.54 | 21070 | 0.4804 | 0.4804 | 43860 | *Acinetobacter* phage Abp9 | *Obolenskvirus* |
| MZ618622 | 87.53 | 12777 | 0.2913 | 0.2982 | 42840 | *Acinetobacter* phage Abp95 | *Obolenskvirus* |
| HE806280 | 87.24 | 17233 | 0.3929 | 0.3754 | 45900 | *Acinetobacter* phage AP22 | *Obolenskvirus* |
| MG366114 | 87.13 | 7042 | 0.1606 | 0.1684 | 41820 | *Acinetobacter* phage PBAB08 | *Myoviridae* |
| MH853786 | 86.99 | 11154 | 0.2543 | 0.2485 | 44880 | *Acinetobacter* phage vB_AbaM_IME285 | *Obolenskvirus* |
| KJ817802 | 86.61 | 11909 | 0.2715 | 0.2715 | 43860 | *Acinetobacter* phage YMC-13-01-C62 | *Obolenskvirus* |
| ON237674 | 86.43 | 18503 | 0.4219 | 0.4123 | 44880 | *Acinetobacter* phage Arbor | *Obolenskvirus* |
| OP585104 | 85.73 | 16683 | 0.3804 | 0.3804 | 43860 | *Acinetobacter* phage vB_AbaM_BP10 | *Obolenskvirus* |
| JX560521 | 70.87 | 12531 | 0.2857 | 0.2925 | 42840 | *Acinetobacter* phage phiAC-1 | *Myoviridae* |
| JF974295 | 67.60 | 525 | 0.0120 | 0.0129 | 40800 | *Psychrobacter* phage pOW20-A | *Myoviridae* |
| MN166823 | 66.00 | 564 | 0.0129 | 0.0221 | 25500 | *Klebsiella* phage ST512-KPC3phi13.5 | *Myoviridae* |
| MT664721 | 65.90 | 913 | 0.0208 | 0.0256 | 35700 | *Escherichia* phage vB_EcoM_APEC | *Podoviridae* |
| MN856093 | 60.50 | 721 | 0.0164 | 0.0544 | 13260 | *Myoviridae* sp. | *Myoviridae* |
| AP014629 | 59.10 | 746 | 0.0170 | 0.0174 | 42840 | *Edwardsiella* phage GF-2 | *Gofduovirus* |
| MH898687 | 59.00 | 746 | 0.0170 | 0.0183 | 40800 | *Edwardsiella* phage Edno5 | *Gofduovirus* |
| MN856055 | 58.70 | 778 | 0.0177 | 0.0196 | 39780 | *Myoviridae* sp. | *Myoviridae* |
| LR798197 | 58.60 | 609 | 0.0139 | 0.0146 | 41820 | uncultured *Caudovirales* phage | *environmental samples* |
| MN855957 | 58.50 | 730 | 0.0166 | 0.0377 | 19380 | *Myoviridae* sp. | *Myoviridae* |
| MN856004 | 58.13 | 2163 | 0.0493 | 0.0505 | 42840 | *Myoviridae* sp. | *Myoviridae* |
| MN855951 | 58.00 | 745 | 0.0170 | 0.0913 | 8160 | *Siphoviridae* sp. | *Siphoviridae* |
| MG592584 | 57.30 | 627 | 0.0143 | 0.0143 | 43860 | *Vibrio* phage 1.214.O._10N.222.54.F11 | *Siphoviridae* |
| MG592398 | 56.90 | 630 | 0.0144 | 0.0144 | 43860 | *Vibrio* phage 1.013.O._10N.286.54.F9 | *Siphoviridae* |
| MN856109 | 55.90 | 759 | 0.0173 | 0.1063 | 7140 | *Myoviridae* sp. | *Myoviridae* |
