# Supplementary figures and images for: New Obolenskvirus Phages Brutus and Scipio: Biology, Evolution, and Phage-Host Interaction
Source: Int J Mol Sci. 2024 Feb 8;25(4):2074. doi: 10.3390/ijms25042074 (PMC10888812; doi:10.3390/ijms25042074)

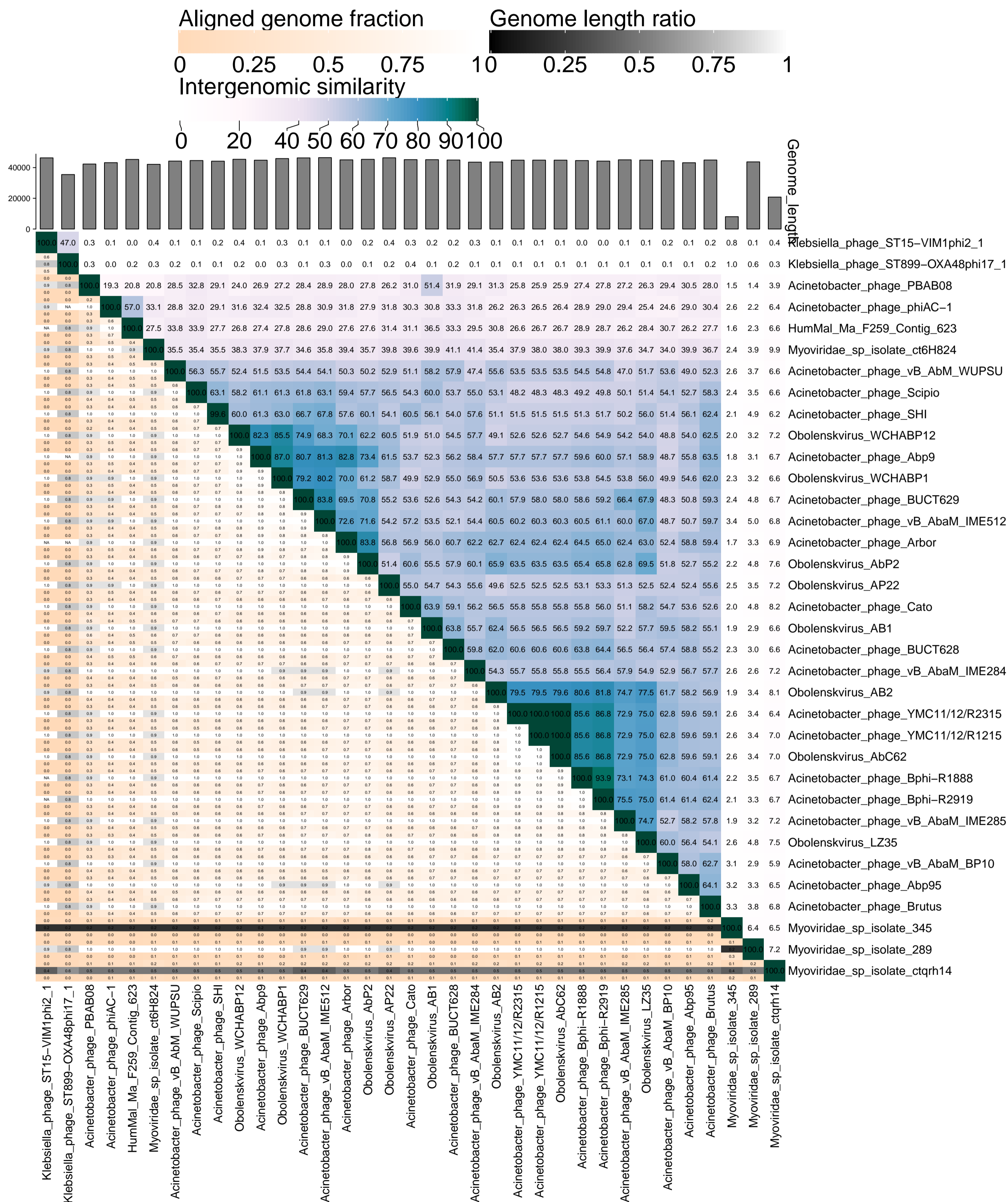

Supplement: Supplementary file 1 [file ijms-25-02074-s001.zip › Supplementary materials_Figure_S1.pdf]
